# Supplementary material for: Is Working from Home during COVID-19 Associated with Increased Sports Participation? Contexts of Sports, Sports Location and Socioeconomic Inequality
Source: Int J Environ Res Public Health. 2022 Aug 14;19(16):10027. doi: 10.3390/ijerph191610027 (PMC9408749; doi:10.3390/ijerph191610027)
Supplement: Supplementary file 1 [file ijerph-19-10027-s001.zip › ijerph-1807972-supplementary.pdf]

Supplementary materials

Table S1a. Adjusted predicted mean MCA estimates in percentages and adjusted ANOVA significance levels for males

		**Sports participation**			**Context of participation**			**Location of participation**					
					Individual[a]			At home[a]			Public space[a]		
		Mean		Eta^2	Mean		Eta^2	Mean		Eta^2	Mean		Eta^2
Working from home	Yes	49	***	.168	40	***	.179	12	**	.103	40	***	.175
	No	43			30			9			33		
Educational level	Lower	28	***	.286	20	***	.236	6	**	.173	19	***	.268
	Middle	40			32			7			31		
	Higher	58			44			16			47		
Economic deprivation	Yes	31	**	.121	28		.073	11		.009	23	*	.115
	No	47			36			10			37		
Age group	18 - 34	53	*	.098	34		.030	10		.041	34		.051
	35 - 54	45			34			12			34		
	55+	44			37			8			40		
Having a child ≤ 12	Yes	46		.018	38		.032	9		.003	37		.010
	No	46			35			11			36		
Hampered by COVID-19	Yes	55		.042	40		.013	8		.043	42		.018
	No	45			35			11			35		
Weekly working hours	8 - 20	44		.018	35		.032	17		.040	36		.008
	21 - 34	49			41			12			39		
	35+	46			34			10			36		
Sufficient sports provision in neighbourhood	Yes	49	***	.166	37	**	.114	11		.036	38	***	.133
	No	31			26			9			26		

N = 732. + $p< 0.10$; * $p < 0.05$; ** $p< 0.01$; *** $p < 0.001$ (two-tailed)

[a] Comparison: Respondents who participated in sports individually/at home/in the public space vs. respondents who did not participate in sports individually/at home/in the public space and those that did not participate in sports at all.

Table S1b. Adjusted predicted meanMCA estimates in percentages and adjusted ANOVA significance levels for females

		**Sports participation**			**Context of participation**			**Location of participation**					
					Individual[a]			At home[a]			Public space[a]		
		Mean		Eta^2	Mean		Eta^2	Mean		Eta^2	Mean		Eta^2
Working from home	Yes	56	***	.203	43	***	.209	19	**	.101	42	***	.211
	No	42			38			15			28		
Educational level	Lower	32	***	.264	21	***	.231	6	***	.177	19	***	.243
	Middle	39			29			15			27		
	Higher	59			43			22			44		
Economic deprivation	Yes	49		.023	30		.058	11		.066	36		.019
	No	49			36			18			35		
Age group	18 - 34	51	*	.093	7		.074	21	*	.108	34		.050
	35 - 54	49			35			17			36		
	55+	47			35			15			34		
Having a child ≤ 12	Yes	40	+	.046	28		.040	13		.041	26	+	.047
	No	51			37			18			37		
Hampered by COVID-19	Yes	53		.004	37		.013	21		.014	33		.037
	No	48			35			17			35		
Weekly working hours	8 - 20	51	*	.130	35	*	.113	18	*	.124	34	*	.105
	21 - 34	46			34			15			35		
	35+	52			37			21			35		
Sufficient sports provision in neighbourhood	Yes	50		.045	36	+	.061	17		.003	35		.039
	No	45			30			18			32		

N = 774. + $p < 0.10$; * $p < 0.05$; ** $p < 0.01$; *** $p < 0.001$ (two-tailed)

[a] Comparison: Respondents who participated in sports individually/at home/in the public space vs. respondents who did not participate in sports individually/at home/in the public space and those that did not participate in sports at all.

Table S2. Estimates from logistic regression analysis

		Sports participation			Context of participation			Location of participation					
					Individual[a]			At home[a]			Public space[a]		
		B		SE	B		SE	B		SE	B		SE
Working from home (ref.=no)		.009	*	.004	.014	**	.004	.007		.006	.011	**	.004
Educational level	Lower (ref.)												
	Middle	.544	**	.185	.676	**	.213	.933	*	.385	.703	**	.216
	Higher	1.382	***	.188	1.320	***	.212	1.624	***	.378	1.498	***	.214
Economic deprivation (ref.=no)		-.318		.214	-.341		.234	-.316		.340	-.314		.235
Sex (ref.=female)		-.103		.128	.014		.131	-.556	**	.177	.095		.131
Age group	18 - 34 (ref.)												
	35 - 54	-.156		.150	-.043		.153	-.099		.195	.090		.154
	55+	-.205		.163	.081		.166	-.329		.221	.228		.167
Having child ≤ 12 (ref.=no)		-.205		.146	-.081		.151	-.301		.210	-.173		.153
Hampered by COVID-19 (ref.=no)		.363	*	.178	.185		.183	.040		.256	.133		.185
Weekly working hours	8 - 20 hours (ref.)												
	21 - 34 hours	-.186		.191	.010		.203	-.258		.270	.076		.206
	35+ hours	.011		.195	.047		.206	-.056		.272	.120		.208
Sufficient sports provision in neighbourhood (ref.=no)		.491	**	.152	.415	*	.163	.021		.216	.400	*	.163
Intercept		-1.228	***	.287	-2.062	***	.314	-2.582	***	.476	-2.300	***	.319

Source: LISS (2021). $N = 1506$. + $p < 0.10$; * $p < 0.05$; ** $p < 0.01$; *** $p < 0.001$ (two-tailed)

[a] Comparison: Athletes who sport individually/at home/in the public space v. athletes who did not sport individually/at home/ in the public space *and* individuals that did not sport.
